# Dual Antiplatelet Therapy and Cancer; Balancing between Ischemic and Bleeding Risk: A Narrative Review

**DOI:** 10.3390/jcdd10040135

**Published:** 2023-03-23

**Authors:** Grigorios Tsigkas, Angeliki Vakka, Anastasios Apostolos, Eleni Bousoula, Nikolaos Vythoulkas-Biotis, Eleni-Evangelia Koufou, Georgios Vasilagkos, Ioannis Tsiafoutis, Michalis Hamilos, Adel Aminian, Periklis Davlouros

**Affiliations:** 1 Department of Cardiology, University Hospital of Patras, 265 04 Patras, Greece; aggvakka@outlook.com (A.V.); anastasisapostolos@gmail.com (A.A.); vbnikos@gmail.com (N.V.-B.); elenikoufou17@gmail.com (E.-E.K.); giorgosvasilagkos@gmail.com (G.V.); pdav@med.upatras.gr (P.D.); 2 First Department of Cardiology, Hippocration General Hospital, National and Kapodistrian University of Athens, 157 72 Athens, Greece; 3 Department of Cardiology, Tzaneio General Hospital, 185 36 Piraeus, Greece; ebousoula@gmail.com; 4 First Department of Cardiology, Red Cross Hospital, 115 26 Athens, Greece; tsiafoutisg@yahoo.com; 5 Department of Cardiology, Heraklion University Hospital, 715 00 Heraklion, Crete, Greece; mich.hamilos@gmail.com; 6 Department of Cardiology, Centre Hospitalier Universitaire de Charleroi, 6042 Charleroi, Belgium; adaminian@hotmail.com

**Keywords:** cancer, acute coronary syndrome (ACS), percutaneous coronary intervention (PCI), dual antiplatelet therapy (DAPT), triple antithrombotic therapy (TAT), atrial fibrillation (AF), cardiotoxicity

## Abstract

Cardiovascular (CV) events in patients with cancer can be caused by concomitant CV risk factors, cancer itself, and anticancer therapy. Since malignancy can dysregulate the hemostatic system, predisposing cancer patients to both thrombosis and hemorrhage, the administration of dual antiplatelet therapy (DAPT) to patients with cancer who suffer from acute coronary syndrome (ACS) or undergo percutaneous coronary intervention (PCI) is a clinical challenge to cardiologists. Apart from PCI and ACS, other structural interventions, such as TAVR, PFO-ASD closure, and LAA occlusion, and non-cardiac diseases, such as PAD and CVAs, may require DAPT. The aim of the present review is to review the current literature on the optimal antiplatelet therapy and duration of DAPT for oncologic patients, in order to reduce both the ischemic and bleeding risk in this high-risk population.

## 1. Introduction

Patients with cancer show a high prevalence of coronary artery disease (CAD) [1]. These diseases share common predisposing factors, such as obesity, diet, sedentary lifestyle, smoking, alcohol, and chronic inflammation [2]. Moreover, cancer itself increases the risk of cardiovascular disease by invading the cardiovascular system directly, releasing metabolites and cytokines, and leading to neurohormonal activation [3,4]. At the same time, anticancer therapies promote inflammation, vasospasm, endothelial dysfunction, plaque formation, and dysregulation of the hemostatic system [5,6].

Patients with cancer who have undergone percutaneous coronary intervention (PCI) and/or suffered an acute coronary syndrome (ACS) may need to discontinue dual antiplatelet therapy (DAPT) due to re-initiation of anticancer therapy, surgery, or biopsies [7]. In addition, since cancer can cause disorders in the hemostatic system, leading to both thrombosis and hemorrhage [6,8], deciding the optimal duration of DAPT in patients with cancer is challenging.

The aim of this study is to review the current literature on the optimal antiplatelet therapy and duration of DAPT for oncologic patients, in order to reduce both the ischemic and bleeding risk in this high-risk population.

## 2. Materials and Methods

A literature review was performed by searching the PubMed database for studies published in the English language up to January 2023. The following key words and their abbreviations were used: “cancer” OR “malignancy” OR “anticancer therapy” AND “dual antiplatelet therapy” OR “acute coronary syndrome” OR “percutaneous coronary intervention” OR “coagulation” OR “cardiotoxicity” OR “transcatheter aortic valve replacement” OR “patent foramen ovale—atrial septal defect closure” OR “left atrial appendage occlusion”. Clinical guidelines, meta-analyses, systematic reviews, retrospective and prospective studies, narrative reviews, and case reports were included. Non-English-language articles and articles with unavailable full text were excluded from further analysis. The articles were considered eligible regarding their clinical relevance to the optimal agents and duration of DAPT in patients with cancer when DAPT is needed.

## 3. Biological and Clinical Aspects of Coagulation in Patients with Cancer

Malignancy may dysregulate hemostatic mechanisms, predisposing cancer patients to both thrombosis and hemorrhage [6]. Approximately 15% of patients with acute coronary syndrome (ACS) have concomitant cancer [7], including lung, prostate, gastric, pancreatic, and breast cancer [9]. The risk of venous thromboembolism (VTE) is fourfold to sevenfold higher in patients with active cancer [10], while approximately 10% of patients with solid cancer experience bleeding, and this incidence is even higher in patients with hematologic malignancies [6,11]. Moreover, according to the study of Guo et al., cancer patients who undergo PCI have a higher risk of thrombotic and ischemic events as well as bleeding after the procedure [12].

Thromboembolic events, which include arterial and venous thrombosis, thrombotic microangiopathy, non-bacterial thrombotic endocarditis, and veno-occlusive disease, can lead to ACS and ischemic stroke. Regarding bleeding, a fatal or a major bleeding event or an ongoing low-degree emission may happen, and it can be manifested either as a localized injury due to tumor invasion or as generalized bleeding predisposition [6,13]

These thromboembolic and bleeding manifestations, which are caused by the dysregulation of the hemostatic system provoked by the cancer, have been associated with both clinical and biological risk factors.

**Figure 1:**
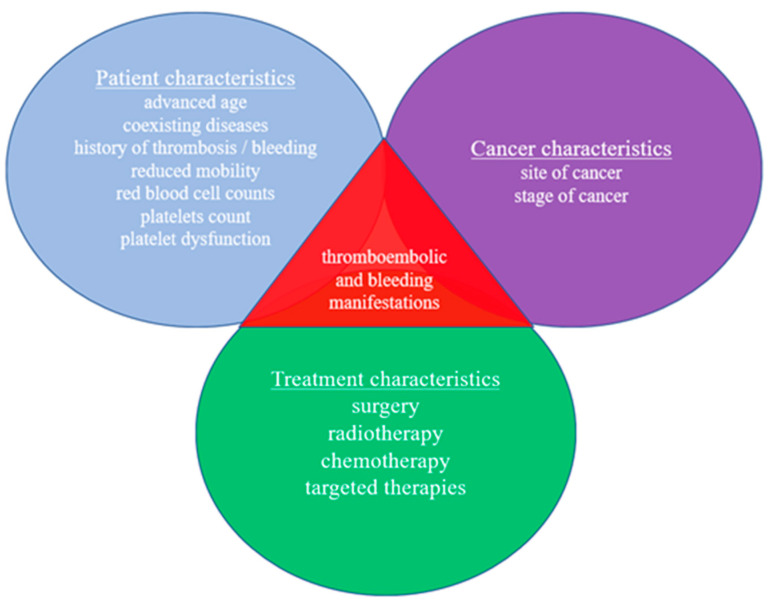
Clinical risk factors for both thromboembolic and bleeding manifestations in patients with cancer.

The clinical risk factors can be divided in three groups, regarding patient characteristics, cancer characteristics, and treatment characteristics [6,14,15,16], as shown in Figure 1. Concerning cancer characteristics, the incidence of VTE is higher in patients with pancreatic, gastric, and lung cancer; hematologic malignancies; and metastatic disease [17]. Malignancies that often cause bleeding include head and neck, lung, gastrointestinal, colorectal, and gynecologic malignancies; acute myelogenous leukemia; chronic lymphocytic leukemia; non-Hodgkin lymphoma; multiple myeloma; Waldenström’s macroglobulinemia; and monoclonal gammopathy of unknown significance (MGUS) [18,19,20]. Patients with active lung cancer or colon cancer treated with PCI are more likely to have a 90-day readmission for acute myocardial infarction (AMI) after PCI, while patients with active colon cancer or metastatic cancer are more likely to have a 90-day readmission for bleeding after PCI [21].

As for biological factors, cancer cells can activate the hemostatic system by expressing and releasing molecules. Specifically, tumor cells activate the coagulation cascade by releasing procoagulant tissue factor, inflammatory cytokines, and microparticles, while they also activate endothelial cells, leukocytes, and platelets by expressing procoagulant proteins and releasing soluble factors [6,8].

Apart from the anticancer therapies, other causes of bleeding in oncologic patients are decreased synthesis of coagulation factors, vitamin K deficiency, excessive fibrinolysis, medication—such as anticoagulation and non-steroidal anti-inflammatory drugs—disseminated intravascular coagulation syndrome (DIC), acquired hemophilia, and acquired von Willebrand Disease [13,18,19].

## 4. Cardiotoxicity Caused by Anticancer Treatment

Damage to the cardiovascular system can be caused by radiation treatment, chemotherapeutic agents, immunotherapies, and targeted therapies (Table 1). The mechanism by which anticancer treatment causes harm varies depending on the agent used [7].

Radiation treatment can cause endothelial injury, accelerated atherosclerosis, plaque rupture, and platelet aggregation since radiation produces free radicals, leading to oxidative stress, DNA damage, and inflammation [20,22].

Additionally, 5-fluorouracil is a chemotherapeutic drug most frequently used in breast, pancreatic, gastric, and colorectal cancer. A systematic review showed that patients treated with 5-fluorouracil developed chest pain as a result of myocardial infarction with ST elevation, most commonly during the first 2 days after administration [23]. The underlying mechanism is considered to be endothelium damage, which promotes inflammation, vasospasm, and plaque formation [24].

Another well-known chemotherapeutic agent, which is used mostly in patients with ovarian, testicular, or small cell lung cancer, is cisplatin. Long-term cardiac events may be related to LDL–HDL imbalance and endovascular damage caused by lipid peroxidation that causes platelet aggregation and thus thrombosis. In acute setting, cisplatin administration has been associated with vasospastic angina [25,26].

Bevacizumab is an anti-VEGF agent used as a first-line therapy for colorectal, lung, breast, and renal cancer. Ischemic heart disease is developed in one per 100 patients treated with bevacizumab and is developed due to endothelium dysfunction [27], while it has also be linked with hemorrhage and arterial thromboembolism [28].

Men with prostate cancer undergoing androgen deprivation therapy and women with breast cancer treated with aromatase inhibitors present an increased risk of cardiovascular events [29,30].

Tyrosine kinase inhibitors (TKIs), such as sorafenib and sunitinib, are both related with hypertension and ACS due to coronary vasospasm because TKIs decrease the vasodilator nitric oxide and increase the vasoconstrictor endothelin-1 [31]. Patients treated with nilotinib and ponatinib develop acute coronary occlusion and myocardial infarction due to progression of atherosclerosis [32].

Immune checkpoint inhibitors (monoclonal antibodies that block the immune brakers or regulators), such as ipilimumab (cytotoxic T lymphocyte-associated antigen-4 inhibitor), nivolumab (programmed death-1 inhibitor), and atezolizumab (programmed death-ligand 1 inhibitor), are related to major cardiovascular events such as myocardial infarction due to the acceleration of atherosclerosis and plaque rupture [33].

The use of the immunomodulatory drugs lenalidomide and pormalidomide in patients with multiple myeloma is associated with increased risk of ACS, but the underlying mechanism needs further investigation [34].

Ibrutinib, which reduces mortality in several B-cell malignancies and chronic lymphocytic leukemia, is associated with atrial fibrillation and increased bleeding risk [35].

**Table 1:** Agents associated with cardiovascular dysfunction.

Treatment	Incidence	Mechanism
Radiation [22]	Depends on the prescribed dose and the cardiac radiation exposure	Endothelial injury, acceleration of CAD, ACS
5-Fluorouracil [23,24]	2–18%	ACS, vasospasm
Cisplatin [25,26]	0.2–12%	ACS, acute thrombosis, acceleration of CAD
Bevacizumab [28,36]	0.52–1.7%	ACS, acute thrombosis
Leuprolide (GNRH agonist) [37]	2.6–5.6%	Angina, ACS, acceleration of CAD
Anastrozole (aromatase inhibitor) [29]	2%	ACS
Tyrosine kinase inhibitors:		
Sorafenib [31]	1%	Acute thrombosis
Sunitinib [31]	5–8%	Acute thrombosis, acceleration of CAD
Nilotinib [20,32]	8–12%	ACS, acceleration of CAD, AF
Ponatinib [32]	2%	ACS, acceleration of CAD
Ibrutinib [35]	8.8%	Bleeding diathesis, AF

ACS, acute coronary syndrome; CAD, coronary artery disease; AF, atrial fibrillation.

## 5. DAPT in Patients with Cancer Undergoing Elective PCI

Since cancer patients undergoing elective PCI have an increased ischemic and bleeding risk, the appropriate antiplatelet therapy remains a challenge. Clopidogrel is the main P2Y12 inhibitor used in these patients since prasugrel and ticagrelor have been associated with more bleeding events, and there are no data in the literature regarding their safety in cancer patients [38].

New technologies entering our quiver, such as new generation drug-eluted stents (DESs), have led to the possibility of shortening the DAPT duration to a minimum of 1 month [39]. After the first month, there is the possibility of extending DAPT up to 3–6 months depending on the patient’s ischemic and bleeding risk, the type and stage of cancer, the need for surgery, and the current cancer treatment [7,20,40]. In their study of 75 patients undergoing PCI with drug-eluting stents (DESs), Balanescu et al. reported that the discontinuation of DAPT at 6 months after DES implantation did not increase the incidence of in-stent thrombosis and restenosis [41]. The shortening of DAPT was feasible and safe using newer generation DESs; thus, cancer therapies with high bleeding risk can be administered more quickly, resulting in potential survival benefits. The authors suggested that cancer therapies can be safely started again at <6 months and as early as 2 weeks after PCI with DESs. However, a retrospective, observational study comparing the outcomes of using bare metal stents (BMSs) or DESs in cancer patients with CAD did not show any significant difference between the number of revascularizations nor the all-cause mortality between cancer patients with CAD treated with BMSs versus DESs during a follow-up period of 34.1 months [42].

Finally, an alternative strategy for these high-risk patients to allow early DAPT discontinuation could be the evaluation of the coverage of the stent’s struts with optical coherence tomography (OCT) [43,44]. In the PROTECT-OCT study, cancer patients who had a recent DES placement (1–12 months) and had to discontinue DAPT prematurely were evaluated using coronary angiograms and OCT [45]. Patients with satisfactory characteristics, such as appropriate stent strut coverage, expansion, apposition, and absence of in-stent restenosis or intraluminal masses, were considered low risk, and DAPT was discontinued, while the remaining patients were considered high risk and stopped DAPT after bridging with low-molecular-weight heparin. In a total of 40 patients, no cardiovascular event occurred in the low-risk group, and only one myocardial infarction occurred in the high-risk group, suggesting that the use of OCT could be useful in the management of this group of patients [45]. Nevertheless, further studies with more patients are required to exact more reliable conclusions.

However, the decision between optimal medical therapy and invasive therapy should be individualized, taking into consideration the cancer prognosis, type of cancer, cancer treatment, and patients’ ischemic and bleeding risks, and it should be made after an extensive discussion between the various specialties involved [46].

## 6. DAPT in Patients with ACS and Cancer

Available data concerning ACS management among cancer patients are limited, making the clinical decision a challenge. Generally, treatment should be personalized according to the ACS subtype, the stage and type of cancer, and the patient prognosis [47], and cancer therapy should be temporarily interrupted, especially if a causal relationship is suspected [48].

The management of patients with cancer presenting with an ACS often requires a multidisciplinary and individualized approach [48]. An invasive strategy should be preferred in ST elevation myocardial infarction (STEMI) patients, as well as in NSTEMI patients who are unstable or are considered high risk. The use of third-generation DESs is indicated because of their lower risk of thrombosis and the need for a shorter duration of DAPT. On the contrary, in clinically stable NSTEMI patients, a conservative non-invasive strategy could be adopted, especially in the case of poor life expectancy and/or of a high risk of bleeding, such as patients with metastases, coagulopathies, or thrombocytopenia [49].

**Figure 2:**
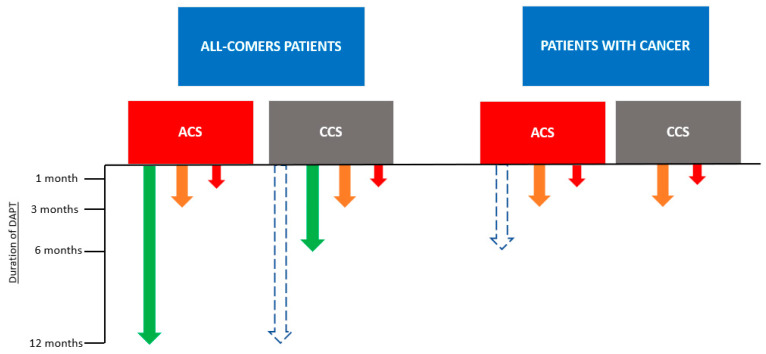
Strategies regarding the duration of DAPT after elective PCI and ACS in all-comers and cancer patients.

DAPT required after PCI poses a great concern in cancer patients, limiting the use of an invasive strategy. DAPT consisting of aspirin and clopidogrel is recommended in these patients, especially in cancer patients with a recent diagnosis (<1 year) or other coexisting bleeding risk factors [10,17]. On the contrary, newer P2Y12 antagonists, such as ticagrelor and prasugrel, should be avoided due to their high bleeding risk and the lack of data on this patient subset. However, ticagrelor or prasugrel may be used under strict surveillance of the bleeding risk in specific patients with previous stent thrombosis during treatment with clopidogrel [48]. According to the 2022 ESC Guidelines on cardio-oncology, the duration of DAPT should be as short as possible, with 1–3 months being proposed as the optimal duration [49,50,51]. If urgent surgery is necessary, interruption of clopidogrel is recommended, as in non-cancer patients [48]. We suggest that a 6-month DAPT may be considered in specific patients with cancer and ACS who are of high ischemic risk, according to risk criteria for extended treatment with a second antithrombotic agent in “2020 ESC Guidelines for the Management of Acute Coronary Syndromes in Patients Presenting without Persistent ST-Segment Elevation” [52] under careful monitoring (Figure 2).

In Figure 2, for all-comer patients, green indicates a low bleeding risk according to “2019 ESC Guidelines for the Diagnosis and Management of Chronic Coronary Syndromes” [53] and “2020 ESC Guidelines for the Management of Acute Coronary Syndromes in Patients Presenting without Persistent ST-Segment Elevation” [52]. Orange indicates a high bleeding risk according to “2019 ESC Guidelines for the Diagnosis and Management of Chronic Coronary Syndromes” [53] and “2020 ESC Guidelines for the Management of Acute Coronary Syndromes in Patients Presenting without Persistent ST-Segment Elevation” [52]. Red indicates a very high bleeding risk according to “2019 ESC Guidelines for the Diagnosis and Management of Chronic Coronary Syndromes” [53] and “2020 ESC Guidelines for the Management of Acute Coronary Syndromes in Patients Presenting without Persistent ST-Segment Elevation” [52]. The dashed blue arrow indicates that in patients with high thrombotic risk and CCS (as described in “2020 ESC Guidelines for the Management of Acute Coronary Syndromes in Patients Presenting without Persistent ST-Segment Elevation” [52]), 12-month DAPT could be considered.

In Figure 2, for patients with cancer, orange indicates active malignancy (excluding non-melanoma skin cancer) within the past 12 months without any other bleeding risk factors. Red indicates active malignancy (excluding non-melanoma skin cancer) within the past 12 months plus at least one major or two minor criteria for high bleeding risk according to the Academic Research Consortium for High Bleeding Risk at the time of percutaneous coronary intervention [52], or according to criteria in “2022 ESC Guidelines on Cardio-Oncology Developed in Collaboration with the European Hematology Association (EHA), the European Society for Therapeutic Radiology and Oncology (ESTRO) and the International Cardio-Oncology Society (IC-OS)” [20], namely: a high risk of gastrointestinal or genitourinary bleeding, significant drug–drug interactions, severe renal dysfunction (creatinine clearance < 30 mL/min), significant liver disease (alanine aminotransferase/aspartate aminotransferase > 2 × ULN), or significant thrombocytopenia (platelet count < 50,000/μL). The dashed blue arrow indicates that in patients with active malignancy and ACS who are of high ischemic risk (as described in “2020 ESC Guidelines for the Management of Acute Coronary Syndromes in Patients Presenting without Persistent ST-Segment Elevation” [52]), 6-month DAPT could be considered. ACS stands for acute coronary syndrome, and CCS stands for chronic coronary syndrome.

**Figure 3:**
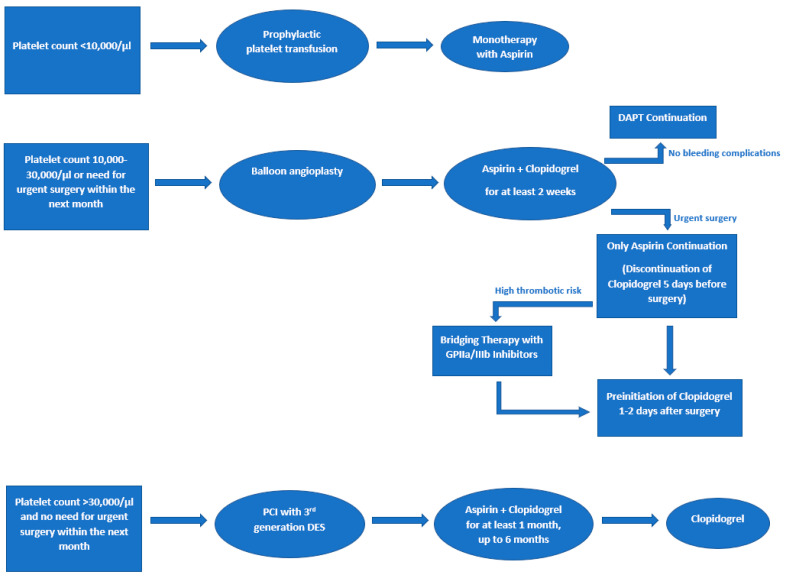
Modified algorithm of Radmilovic et al. for the management of antiplatelet therapy in cancer patients. DES = drug-eluting stent, DAPT = dual antiplatelet therapy, PCI = percutaneous coronary intervention.

Furthermore, the platelet count should be taken into consideration when DAPT is administered in patients with cancer. Aspirin is allowed if the platelet count is >10,000/μL, while DAPT initiation (with aspirin and clopidogrel) is allowed if the platelet count is >30,000/μL [7,54]. Ticagrelor, prasugrel, and glycoprotein IIb/IIIa inhibitors should be used with more caution in cancer patients and should be avoided in patients with a platelet count <50,000/μL/ [55]. In addition, if the platelet count is <20,000/µL, prophylactic platelet transfusion may be considered [56]. Taking into account the platelet count and the need for urgent surgery or chemotherapy, Radmilovic et al. also suggested a protocol [7] regarding the management of antiplatelet therapy in cancer patients taking into account the platelet count (Figure 3).

Bleeding in patients with ACS increases mortality and requires clinical decisions on the continuation of DAPT. Bleeding could lead to anemia, which is an independent risk factor for ACS [57]. The severity of bleeding should be taken into consideration when deciding the continuation or discontinuation of antiplatelet therapy. In this way, DAPT can be maintained in cases of minor bleeding (such as hematomas). On the contrary, DAPT should be stopped in cases of severe bleeding (such as need for hospitalization or >2g/dL decrease in hemoglobin levels), and monotherapy with clopidogrel should be considered thereafter. In the case of life-threatening bleeding, all antiplatelet agents should be discontinued [7].

In many cancer patients, comorbidities such as atrial fibrillation (AF), venous thromboembolism, and valvular heart disease often coexist. However, triple antithrombotic therapy (TAT), which would be indicated in the absence of cancer, is not advised because of the significantly higher risk of bleeding [49]. Thus, the administration of a novel oral anticoagulant (NOAC) and a single oral antiplatelet agent (preferably clopidogrel) is preferred after a short period of triple antithrombotic therapy (TAT) (up to 1 week in the hospital) [48].

It should be noted that several scores (such as PARIS and DAPT) used to assess the bleeding risk regarding the duration of antiplatelet therapy after PCI have not been validated in patients with malignancy [49], while the PRECISE-DAPT score did not perform well for predicting bleeding in oncologic patients [58]. In addition, cancer has not been included in the most common risk scores, such as CHA2DS2-VASc and HAS-BLED, making the clinical decision of balancing the higher ischemic and bleeding risk even more difficult [49]. A risk assessment model (RAM) for VTE that is applicable to patients with specific types of solid tumor after the initiation of anticancer therapy is the COMPASS–CAT RAM [59].

Although platelet function testing (PFT) is not performed on a routine basis in patients with ACS or stable CAD, it may be a useful tool for guiding antiplatelet treatment escalation in patients with high platelet reactivity (HPR) on clopidogrel and screening for HPR on clopidogrel when DAPT de-escalation is necessary in complex cases [60]. Patients with advanced cancer display platelet hyperreactivity [61], with a higher number of platelets stably adhering to von Willebrand factor (VWF) and greater platelet surface coverage compared with those patients with early-stage cancer [62]. On the contrary, in patients with acute myeloid leukemia (AML) and thrombocytopenia, reduced platelet aggregation and platelet activation predict bleeding better than platelet count alone [63]. Thus, since platelet function is altered in patients with cancer, PFT may be useful in adjusting DAPT in oncologic patients, while taking into consideration their ischemic and bleeding risk.

## 7. Antiplatelet Therapy in Patients with Cancer Undergoing CABG

As complex PCI has become a reasonable and safe choice in daily practice in the majority of catheterization laboratories, the overall utilization of coronary artery bypass grafting (CABG) has decreased over time (250,677 in 2003 vs. 134,534 in 2015), while the proportion of those with comorbid cancer undergoing CABG has increased (7% vs. 12.6%, *p* < 0.001) [64].

Although most cancer patients with CAD are treated conservatively or with PCI, given the prevalence of complex coronary disease and the potential challenges of prolonged anticoagulation therapy in the presence of cancer, CABG may be sometimes the best option for these patients, mainly for those without active cancer or those with >1 year life expectancy [20]. Moreover, according to Guha et al., the presence of breast, lung, prostate, and colon cancer and lymphoma does not appear to be associated with increased in-hospital mortality in cancer versus non-cancer patients with CABG. However, there is a higher bleeding risk in CABG patients with breast and prostate cancer compared with non-cancer patients with CABG [64]. Even in non-cancer patients undergoing CABG, guidelines and clinical practice are not uniform and specific regarding DAPT therapy, especially in the setting of chronic coronary syndrome (CCS). According to the latest guidelines of the European Association for Cardio-Thoracic Surgery [65] and the American Heart Association [66], there is limited evidence regarding DAPT after CABG in CCS. Therefore, based on extensive evidence, aspirin is strongly recommended (class IA recommendation) for all patients after CABG by both American and European guidelines, whereas the use of DAPT in CABG patients without a separate indication (e.g., ACS) is graded as class IIb, meaning they only provide some benefit [67,68]. Thus, cancer patients undergoing CABG for CCS should be treated with a single antiplatelet agent.

Many patients with malignancies suffer from atrial fibrillation (AF) and/or VTE for which they should take anticoagulants. For this population undergoing CABG, a short course of combined antithrombotic therapy with an antiplatelet and an NOAC is recommended, followed by monotherapy with an NOAC lifelong [65].

## 8. Antithrombotic Therapy in Patients with AF and Cancer Undergoing PCI or Suffering from ACS

Different cancer types are associated with different bleeding risk profiles. Active cancers (especially hematologic malignancies and gastrointestinal cancers) and existing metastases increase the bleeding risk [69]. Moreover, cancer patients have a higher rate of bleeding after PCI compared with non-cancer patients [12]. Taking this into consideration, along with the fact that cancer patients with AF are already under anticoagulation therapy, it is preferred to keep DAPT as short as possible in cancer patients with AF after stent implantation or ACS.

According to the 2022 ESC Guidelines on Cardio-Oncology, when both anticoagulation and antiplatelet therapy are needed, TAT can be administered for a short period of time (up to 1 week in the hospital), and then an NOAC and single oral antiplatelet agent (preferably clopidogrel) is the default strategy [20]. The combination of NOACs plus a P2Y12 inhibitor was associated with less bleeding without a significant difference in major adverse cardiovascular events (MACE), compared with the use of vitamin K antagonists (VKA) plus DAPT [70,71,72,73]. A combination of VKA plus DAPT should be avoided due to dramatically increasing bleeding complications [72].

In patients treated with oral anticoagulants (OACs) undergoing PCI, bleeding complications occur mostly in the first period of treatment, and this risk remains elevated over time [74,75]. Therefore, in patients with additional risk factors for bleeding, the duration of aspirin therapy should not exceed the peri-PCI period, namely, during inpatient stay, until the time of discharge [74].

Clopidogrel is the most studied P2Y12 inhibitor in (≈88%) patients enrolled in trials of AF patients treated with an NOAC undergoing PCI [76,77,78,79,80]. Prasugrel should not be used concomitantly with an OAC, while ticagrelor may be a good alternative to clopidogrel for specific cases of cancer patients [74]. Considering that cancer patients treated with an OAC are at a high risk of bleeding, the duration of the P2Y12 inhibitor administration should be as short as possible after PCI or ACS, and then patients should continue with the OAC at the appropriate dose. Whether the P2Y12 inhibitor would be discontinued after 1, 3, or 6 months or in between probably depends on the specific profile of the patient and is up to the discretion of the treating physicians [74].

## 9. Antiplatelet Therapy in Patients with Cancer Undergoing Cardiac Structural Interventions

Apart from PCI, there are also other structural interventions, such transcatheter aortic valve replacement (TAVR), patent foramen ovale (PFO), or atrial septal defect (ASD) closure and left atrial appendage (LAA) occlusion, which include device implantation and require the appropriate antiplatelet therapy as a prevention measure for thrombosis.

### 9.1. TAVR

Nowadays, TAVR has gained significant ground in the management of aortic stenosis. While it was applied mainly in very high-risk patients, recent data support that TAVR is a feasible and safe option even for low-risk patients [81,82]. Moreover, TAVR is indicated for oncologic patients with active cancer or cancer in remission, as the existing literature supports that TAVR should be preferred when compared with medical treatment or surgical replacement [83,84,85].

The optimal antiplatelet post-TAVR therapy remains under investigation [86]. Capodanno and colleagues suggested that single-antiplatelet treatment (SAPT) should be administered in post-TAVR patients without any indication for DAPT. Aspirin should be preferred, whereas clopidogrel could be the alternative option [87]. The only indication in which DAPT should be chosen is for patients who need DAPT for another reason, such as coronary stenting during the last 3 months. In these patients, an individualized approach should be followed, and DAPT could be administered for no more than 6 months; then, it should be replaced with SAPT [88]. On the contrary, the recent OCEAN-TAVI Registry showed that the nonantithrombotic strategy after TAVR does not increase the risk of net adverse clinical events and reduces the bleeding risk in patients who do not need anticoagulation therapy [89], leading to the conclusion that a nonantithrombotic approach after TAVR may be feasible in specific oncologic patients with high bleeding risk. However, future studies are required to establish the necessity, duration, and agents of antiplatelet therapy after TAVR in patients with cancer.

### 9.2. PFO-ASD Closure

Thanks to the progress of interventional cardiology, transcatheter PFO and ASD occlusion have been established as feasible and safe procedures. After the procedure and until complete endothelization of the device, DAPT is required mainly for thrombosis prevention and secondarily for nickel release inhibition. Endothelization is estimated to be completed in 3–6 months, so the duration of DAPT should be adapted respectively [90,91,92]. Regarding the duration of DAPT in this field, the existing literature lacks large-scale, randomized trials to provide adequate data; current practice is established on based consensus statements and empirical approaches. Recently, Pristipino et al. published the first European position on the management of patients with PFO [93]. Based on current studies, the experts advised using DAPT for 1–6 months (strength: conditional, evidence level: A), which should be followed by SAPT with aspirin for at least 5 years (strength: conditional, evidence level: C) [94,95]. Interatrial shunt closure in the setting of active cancer remains poorly investigated. Taking into consideration the thrombogenicity of malignancies, percutaneous PFO closure could theoretically be beneficial acting as a protective shield against thrombus formation and embolization to cerebral circulation. Further studies are required to evaluate the benefit/harm ratio in patients with active malignancy undergoing PFO closure as a secondary prevention strategy [96].

### 9.3. LAA Occlusion

Fatal strokes are the main mortality cause in patients with AF, while the emboli are created in the LAA. Studies have reported that surgical LAA occlusion has been associated with reduced incidence of both fatal and non-fatal strokes. Notably, cancer patients who need anticoagulation therapy due to AF are commonly candidates for this specific intervention due to their high bleeding and ischemic risk [97]. However, no common line exists regarding the most appropriate antiplatelet therapy for either patients with cancer or generally people undergoing LAA closure. Chen et al. supported that either short-term DAPT for 6 weeks or SAPT should be preferred due to the hemorrhagic risk in people undergoing LAA closure [98]. A newer study showed that SAPT or even no therapy does not increase the ischemic risk [99], while a recent, non-randomized study found that SAPT instead of DAPT after LAA occlusion was associated with a reduction of bleeding complications, with no significant increase in the risk of thrombotic events [100]. Therefore, a tailored approach should be followed, pending for large-scale, suitably designed clinical trials.

## 10. Antiplatelet Therapy for Non-Cardiac Diseases in Patients with Cancer

Antiplatelet therapy also plays a pivotal role in non-cardiac diseases, such as peripheral artery disease (PAD) and cerebrovascular accidents (CVAs).

### 10.1. PAD

PAD often coexists with CAD, hypertension, dyslipidemia, and diabetes mellitus. Nowadays, the management of PAD includes percutaneous stent implantation regardless of the location of the lesions. Thanks to the newer drug-eluting stents, carotid artery stenting (CAS) has become a safe and feasible approach with comparable results to surgery. According to the recent ESC Guidelines about PAD [101], stent implantation in the carotid artery should be followed by DAPT (aspirin + clopidogrel) for 1 month (class IA recommendation). After this time frame, DAPT should be replaced with SAPT, with either aspirin or clopidogrel. A similar approach should be followed in patients with lower-extremities artery disease. After percutaneous revascularization, DAPT administration for 30 days is required prior to switching to SAPT (aspirin or clopidogrel). However, the existing literature for lower-extremities artery disease lacks large-scale, randomized studies, so the strength of evidence is limited (class IIa C). To date, special recommendations for cancer patients with concomitant PAD are not available. Thus, application of the guidelines relevant to the general population in this subpopulation should be considered. Nevertheless, a personalized approach based on the ischemic and bleeding risk of each patient should be followed.

### 10.2. CVAs

CVAs remain one of the major causes of mortality and disability globally. The progress of imaging techniques, reperfusion therapy, and improved medical treatment during the last decades has significantly increased the life expectancy of these patients [102]. Although the optimal antithrombotic treatment is important to minimize the incidence of ischemic CVAs, the optimal regimen remains under investigation.

A recent guideline by the European Stroke Organization (ESO) strongly advises the administration of DAPT (aspirin + clopidogrel) for 21 days in patients with a non-cardioembolic minor ischemic stroke or high-risk transient ischemic attack (TIA) during the last 24 h. Moreover, the experts recommend that DAPT (aspirin + ticagrelor) for 30 days may be beneficial in patients with non-cardioembolic mild-to-moderate ischemic stroke or high-risk TIA in the last 24 h [103].

According to a recent meta-analysis of randomized trials, short-term (for up to 3 months) DAPT seems to reduce the risk of recurrent stroke at the expense of a higher risk of major bleeding, compared with aspirin, in patients with high-risk TIA or mild to moderate ischemic strokes [104].

Cancer patients are at higher risk of suffering from acute CVAs and fatal strokes. In particular, patients with prostate, breast, and colorectum malignancies are more prone to fatal strokes [105]. Recently, Bang and colleagues [106] proposed that cancer-related strokes could be an emerging subtype of ischemic stroke, with unique underlying pathophysiological mechanisms. However, the existing literature and current evidence cannot adequately support the precise and tailored antithrombotic management of these patients.

## 11. Conclusions

Since oncologic patients are at high risk for both ischemic and bleeding events due to the dysregulation of their hemostatic system by cancer, the appropriate duration and the optimal agents of antiplatelet therapy after undergoing PCI and/or suffering from an ACS remain a challenge. The use of new technologies, such as DESs and OCT, may lead to shortened DAPT duration in all-comer patients, including patients with cancer. The optimal duration of DAPT is considered to be 1–3 months, consisting of aspirin and clopidogrel, while TAT can only be administered for a short period of time (up to 1 week in the hospital), followed by an NOAC and a single oral antiplatelet agent (preferably clopidogrel). Other structural interventions, such as TAVR, PFO-ASD closure, and LAA occlusion, and non-cardiac diseases, such as PAD and CVA, may require DAPT. Although further studies are needed in order to establish the optimal duration and agents of DAPT, it is indisputable that a personalized and multidisciplinary approach is necessary to increase the life expectancy and quality of life of patients with cancer and CVD, along with finding the balance between thrombotic and bleeding risk.

## Data Availability

Not applicable.
